# Quantitative fluorescence resonance energy transfer-based immunoassay for activated complement C1s

**DOI:** 10.3389/fimmu.2023.1081793

**Published:** 2023-01-24

**Authors:** Jun Ye, Jie Xu, Chuanmeng Zhang, Li Zhu, Sheng Xia

**Affiliations:** ^1^ Department of Immunology, School of Medicine, Jiangsu University, Zhenjiang, Jiangsu, China; ^2^ The Center for Translational Medicine, The Affiliated Taizhou People’s Hospital of Nanjing Medical University, Taizhou, Jiangsu, China

**Keywords:** complement, C1s, FRET, C4, C2, immunoassay

## Abstract

**Objectives:**

C1s activation is associated with the pathogenesis of various diseases, indicating the potential value of C1s activation detection in clinic. Here we aimed to establish fluorescence resonance energy transfer (FRET)-based immunoassay for the quantitative detection of activated C1s in serum.

**Methods:**

FRET-based fluorogenic peptides, sensitive to the enzymatic activity of activated C1s, were prepared and labeled with the fluorophore ortho-aminobenzoic acid (Abz) and quencher 2,4-dinitrophenyl (Dnp), and then were further selected depending on its Kcat/Km value. C1s in the samples was captured and separated using anti-C1s-conjugated magnetic microbeads. Next, enzymatic activity of activated C1s in samples and standards was examined using fluorescent quenched substrate assays. Limit of detection (LOD), accuracy, precision, and specificity of FRET-based immunoassay were also investigated.

**Results:**

This method presented a linear quantification range for the enzymatic activity of activated C1s up to 10 μmol min^-1^ mL^-1^ and LOD of 0.096 μmol·min^-1^·mL^-1^ for serum samples. The recovery of the method was in the range of 90% ~ 110%. All CV values of the intra-analysis and inter-analysis of three levels in samples were less than 10%. The cross-reaction rates with C1r enzyme, MASP1, and MASP2 were less than 0.5%. No significant interferences were found with bilirubin (0.2 mg mL^-1^), Chyle (2000 FTU), and haemoglobin (5 mg mL^-1^), but anticoagulants (EDTA, citrate and heparin) inhibited the enzymatic ability of activated C1s. Thus, this established method can be used for the determination of active C1s in human serum samples in the concentration interval of 0.096-10.000 μmol min^-1^ mL^-1^.

**Conclusions:**

One anti-C1s-based FRET immunoassay for activated C1s detection in serum samples were established, and it will be useful to explore the role of C1s activation in the pathogenesis, diagnosis and treatment in complement-related diseases.

## 1 Introduction

Complements are natural immune molecules that serve as the first line-of-defence in the immune response (1–3). In the physiological state, complement molecules are quiescent. In the adaptive and innate immune states, complement molecules are sequentially activated, which then further promote the immune response. For decades, studies have shown that the activation of the complement system is associated with the onset, progression, and prognosis of various diseases, such as novel coronavirus infection, tumours, and autoimmune diseases (1–4). Up to now, three complement activation pathways have been identified, including the classical pathway, alternative pathway, and mannose-binding lectin pathway. Activation of the complement pathways requires soluble complement molecules, cell membrane receptors, or regulatory molecules. Complement-related biomarkers can be monitored to predict disease progression.

C1 is a multimolecular protease that triggers the activation of the classical pathway, which functions in antimicrobial host defence, immune tolerance, and recognition of abnormal structures. The C1 complex is a Ca^2+^-dependent tetramer, which comprises two copies of two proteases, C1r and C1s, and a recognition protein, C1q (C1qr_2_s_2_) (5). C1q mediates the binding of C1 to the target molecule, thereby inducing the activation of C1r, which converts the proenzyme C1s specifically to cleave C2 and C4 (6). The function of the classical complement system largely depends on the activation of the subcomponent C1s (7). Therefore, quantitative detection of active C1s can help to understand the exact role of classical pathway activation in the pathogenesis of complement-associated diseases.

Indeed, C1s play important roles in maintaining homeostasis and onset of certain diseases. In particular, C1s mutations are associated with rare genetic diseases, infectious susceptibility and autoimmune disorders. Ongoing studies also have indicated that the aberrant activation of C1s contributes to the onset of autoimmune and infectious diseases, and even cancers (8, 9). In recent years, inhibitors and monoclonal antibodies against C1s have been explored in several clinical trials. It is noteworthy that the FDA have approved the C1s monoclonal antibody Sutimlimab (sutimlimab-jome; ENJAYMO™) for the treatment of cold lectin disease (CAD) in February 2022 (10). Therefore, clinical evaluation of C1s activation may serve as a novel potential clinic biomarker especially for diagnosis, prognosis evaluation, and even for the selection of individualized therapies against activated C1s in diseases (11). For the detection of C1s, various methods have been established, including immunohistochemical analysis, immunoblotting (12), immunofluorescence staining (13), bilateral diffusion (14), gelatin zymography (15), LC-MS/MS (16) and ELISA (17). These methods can detect both natural and active complement molecules, but can not effectively distinguish whether C1s are in an active or inactive state. As the complement system is only functional when activated in cascade, it is particularly important to detect whether these complement components are activated or not.

**Figure 1:**
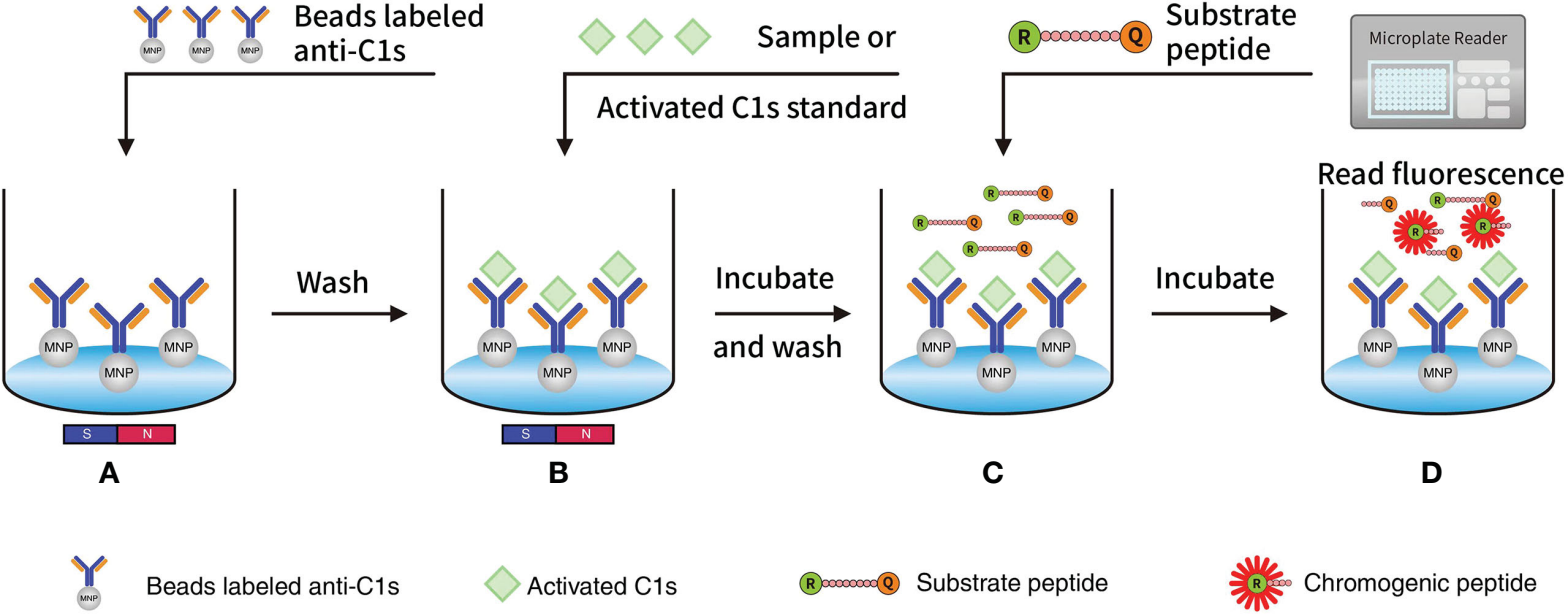
Quantitative FRET immunoassay of the activated C1s based on the capture of anti-C1s antibody: **(A)** MNP or Beads were precoated with antibody against activated C1s and then added into wells; **(B)** Activated C1s standard or samples were added and then specifically combined with MNP-labelled anti-C1s antibody; **(C)** Substrate peptide was added and then cleaved by activated C1s; **(D)** Fluorescence intensity from Abz on the substrate fragment was monitored by a microplate reader, and the enzymatic ability of activated C1s was quantitatively analysed. Abz, ortho-aminobenzoic acid; MNP, magnetic nanoparticles; FRET, fluorescence resonance energy transfer.

Fluorescence resonance energy transfer (FRET)-based immunoassays exploit the distance-dependent transfer of resonance energy from an excited donor fluorophore to a proximal ground-state acceptor fluorophore (18, 19). The spectral properties of the FRET donor-acceptor complex enable direct detection of the acceptor emission without a separation step from the unbound partners (18, 19). The development of homogeneous FRET-based immunoassays contributes to quick and highly specific on-site detection of protein biomarkers for clinical molecular diagnostics of cancers (20, 21) and autoimmune diseases (22). Based on the specific cleavage of C2 and C4 proteins by the active C1s (23), we currently designed the FRET-based peptide that could be cleaved by the active C1s, which conjugated with magnetic microbeads. This study aimed to establish a quantitative immunoassay for the detection of active C1s in serum samples (**Figure 1** **)**.

## 2 Materials and methods

### 2.1 Materials

#### 2.1.1 Reagents and instruments

Buffer solution used in this study included phosphate-buffered saline (PBS) (0.137 M NaCl, 0.0027 M KCl, 0.01 M Na_2_HPO_4_, 0.002 M KH_2_PO_4_, pH 7.4); Tris buffer (0.05 M Tris, 0.15 M NaCl, 0. 2% (w/v) PEG 8000); assay buffer (50 mM Tris, 250 mM NaCl, pH 8.0); binding buffer (0.1 M MES buffer, pH 5.0); washing buffer (TBS with 0.05% Tween 20); and blocking buffer (0.05 M Tris with 1.0% BSA and 0.05% Tween 20). In addition, Tris buffer with 0.05% Tween 20 and BSA was used as the desired buffer. A Synergy HT Multifunction Microplate Reader was purchased from BioTek (Winooski, VT, USA).

Carboxyl-coated magnetic beads (1 μm) were purchased from Merck (Whitehouse Station, NJ, USA). C1s enzyme (A104, activated C1s, double-chain C1s) and C1r enzyme (A102, activated C1r, double-chain C1r) were purchased from CompTech (Tyler, TX, USA). MASP1 (H00005648) and MASP2 (H00010747) were purchased from Abnova (Taipei City, Taiwan, China). C1r, MASP1, and MASP2 were diluted in PBS with concentrations of 660 μg·mL^-1^, 5000 μg·mL^-1^, and 5000 μg·mL^-1^, respectively.

Interference Check A Plus A Kit (ZG0501) was obtained from Sysmex (Kobe, Japan). C1s ELISA assay kit was purchased from RapidBio Systems (Catalog NO: 6141000964; Calabasas, CA, USA). Anti-C1s antibody was screened in-house developed, and then sequenced and genetically engineered with CHO system by Biointron (Taizhou, China). N-Carbobenzyloxy-Lys-thiobenzyl ester (Z-K-SBzl) (M-1300) was purchased from Bachem (Bubendorf, Switzerland). 5,5’-dithio-(bis-2-nitrobenzoic acid) (DTNB) (D-8130) was purchased from Sigma-Aldrich. 1-Ethyl-3-[3-dimethylaminopropyl] carbidedimide hydrochloride (EDC) (22980) was purchased from Thermo Fisher Scientific (Cleveland, OH, USA).

#### 2.1.2 Patients enrolment and sample collection

Total 20 patients with primary diagnosis of rheumatoid arthritis (RA) and 20 healthy individuals matched for sex and age (± 10 years) were enrolled. The diagnosis of RA was in accordance with the 2010 American College of Rheumatology (ACR)-European League Against Rheumatism (EULAR) classification criteria for rheumatoid arthritis.

Venous blood was collected by antecubital venepuncture from participants. Serum and plasma samples treated with different anticoagulants (citrate, EDTA, or heparin) were collected and stored at -80°C before use. This study is complied with all relevant national regulations and institutional policies, and is in accordance with the tenets of the Helsinki Declaration (as revised in 2013). This study has been approved by the Ethics Committee of Taizhou People’s Hospital (KY2020-184-01). Informed consent was obtained from each subject.

### 2.2 Design and preparation of peptide substrates

Substrate candidates were designed based on that active C1s cleave complement components C2 and C4 (24, 25). Three peptide substrates, namely, peptide 1 (GLQRALEI), peptide 2 (SLGRKIQI) and peptide 3 (GYLGRSYKVG) were finally selected for further optimization as previously described (25). The substrate library peptides were synthesized with a purity of greater than 97.43% by Scipeptide (Shanghai, China). Next, FRET-based fluorogenic peptides were prepared with the fluorophore ortho-aminobenzoic acid (Abz) and quencher 2,4-dinitrophenyl (Dnp).

The activity of active C1s on the fluorogenic peptides was firstly studied. Here, 2.5 μL of active C1s (100 μg/mL) (A104, CompTech. Tyler, TX, USA) was added to each well containing the individual peptides with different concentrations (0, 4.72, 9.44, 18.88, 37.75, 75.5, 150, and 300 μM). The reaction system was supplemented with Tris buffer in a volume of 100 μL. Fluorescent intensity was monitored using a microplate reader (BioTek, Winooski, VT, USA) every 5 mins for 2 h at 37°C. The activity of C1s on the individual substrate was monitored based on the alterations of the fluorescence of Abz at excitation wavelength of 360/40 nm and emission wavelength of 460/40 nm. Subsequently, the relationship between the substrate concentration and reactivity was fitted by the Michaelis-Menten equation. V_max_ and K_m_ values of the samples were calculated. Then, K_cat_ was calculated using the formula K_cat_ = V_max_/molecular weight. The peptide with the highest K_cat_/K_m_ value was selected as the candidate substrate for further experiments.

### 2.3 Preparation of anti-C1s antibody-conjugated magnetic microbeads

Firstly, the quality of anti-C1s was characterized by using polyacrylamide gel electrophoresis and size exclusion chromatography-high performance liquid chromatography (SEC-HPLC) (**Supplementary Figure 1**). Moreover, the binding capacities of anti-C1s with active C1s, C1(C1q2C1r2C1s) and C1s zymogen were analyzed using ELISA. The data showed that Anti-C1s had capability to bind all three C1s forms, but it had stronger binding with active C1s than C1 and C1s zymogen. There no difference between C1 and C1s zymoger to bind with anti-C1s(**Supplementary Figure 2**). Next, anti C1s-conjugated magnetic microbeads were prepared using carboxyl-coated magnetic beads and anti-C1s antibody according to the manufacturer’s instructions. Approximately 10 mg magnetic microbeads were washed in binding buffer three times before using. The recombinant anti-C1s monoclonal antibody (200 μg) was mixed with the magnetic microbeads, followed by gentle agitation and incubation for 15 min. Then, 1-ethyl-3-(3-dimethylaminopropyl)-carbodiimide (EDC) was used as the couplant. In detail, 500 μg of the couplant was added into 50 mg microbeads, mixed gently *via* vortexing, and incubated for 1 h. The mixture was next added with 1 mL of binding buffer, and further incubated at room temperature. Next, the beads were washed three times 3 h after the incubation, followed by resuspending in 1 mL of Tris buffer with the desired buffer and storing at 4°C. Before use in the assay, microbeads were washed, magnetically separated and aspirated.

### 2.4 Enzymatic hydrolysis of colorimetric peptide substrates by C1s

The activity of recombinant human complement C1s protein was measured using N-carbobenzyloxy-Lys-thiobenzyl ester (Z-Lys-SBzl) in the presence of 5,5’-dithio-bis(2-nitrobenzoic acid) (DTNB) as described previously (26). The specific enzymatic activity of C1s was calculated as follows:


$$\text{Specific Activity }(pmol/\min/\mu g)=\frac{\text{Adjusted Vmax}⋄(\text{OD}/\text{min})\text{ x well }{\text{volume(L)x10}}^{12}\text{pmol/mol}}{\text{ext}{\text{.coeff}}^{□}{\text{(M}}^{-1}{\text{cm}}^{-1}\text{) x path corr}.^{△}\text{ (cm) x amount of enzyme (μg) }}$$

where ^⋄^ represented adjusted for the substrate blank; ^□^ represented an extinction coefficient of 13260 M_-1_·cm_-1_; and ^△^ represented a path correction of 0.320 cm, respectively.

Our prior data showed that the activity of 1 μg of the C1s standard used in the experiments was 0.0534 μmol/min. Bound with anti-C1s had no obviously negative effects on the enzymatic capacity of active C1s (**Supplementary Figure 3**).

### 2.5 Serum and plasma C1s measured by ELISA

C1s levels in serum and plasma samples were detected using an ELISA kit according to the manufacturer’s instructions. Serum and plasma samples were diluted in dilution buffer. Briefly, 50 μL of human complement C1s standard or diluted samples were added into each well and incubated with 100 μL of horseradish peroxidase (HRP)-labelled C1s antibody. The plate was sealed and incubated at 37°C for 1 h. The microplate was washed three times with wash buffer manually. Then, 100 μL of a mixture of chromogen solution A and solution B was added to each well, and then the plate was incubated at 37°C for 15 min in the dark. The absorbance of each well was read at a wavelength of 450 nm within 15 min after 50 μL of stop solution was added to each well and gently mixed. A standard curve was generated by plotting the absorbance of standards on the X-axis against the concentrations of C1s (0, 5, 10, 20, 40, and 80 μg/mL) on the Y-axis. Then the concentrations of C1s in serum and plasma samples were calculated according to the standard curve.

### 2.6 Fluorescent quenched substrate assays for C1s activity on chromogenic peptides

The enzymatic activity of C1s in the samples was measured. Activated C1s standards or samples were sequentially diluted 10-fold into PBS. then added to the wells (100 μL per well) of a 96-well microtiter plate. A constant amount of anti-C1s antibody-conjugated magnetic microbeads (10 mg/mL, 30 μL per well) were added to each well of the microtiter plate and agitated at room temperature for 10 minutes. The beads were washed with 200 μL of PBS for three times, magnetically separated, and resuspended in 90 μL of Tris buffer. Next, 10 μL of chromogenic peptide (1 μg/mL) was added to each well and mixed adequately. The fluorescence of each well was determined immediately.

### 2.7 Optimal reaction time and standard curve

To determine the optimal reaction time, the reaction was performed with 10 μL of chromogenic peptide 3 (1 μg/mL) and activated C1s with different concentrations (0.625, 1.25, 2.5, 5, and 10 μmol·min^-1^·mL^-1^). The standard curve was prepared with the activated C1s standards (10, 5, 2.5, 1.25, 0.625, and 0 μmol min^-1^ mL^-1^). The activity of the C1s standards was set on the X-axis, and the detected fluorescence intensities were set on the Y-axis. The linear least square method was used to fit these log data to prepare the standard curve, which was used to calculate the C1s enzymatic activity in the samples.

### 2.8 Limit of detection, accuracy, precision, and specificity

To confirm LOD, the reaction was carried out under optimal conditions. The fluorescence intensity was repeatedly examined in the absence of activated C1s. For the accuracy of the optimal method, the relative recoveries were examined. Human serum samples were spiked with activated C1s standard solutions (2.671, 2.671, and 5.342 μmol·min^-1^·mL^-1^) with a volume ratio of 1:1. The relative recovery rate was calculated. The intraday repeatability and interday reproducibility were examined, and the coefficients of variation (CVs) were calculated. For clinical samples, complement analogues in serum, such as C1r, MASP1, and MASP2 may interfere with the detection of C1s activity. Therefore, C1r, MASP1, and MASP2 were assayed for cross reactivity. Then, the cross-reaction rates were calculated using the following formula: the cross-reactivity (%) = (measured concentration/original concentration) × 100%.

### 2.9 Statistical analysis

GraphPad Prism 5.0 (GraphPad Software, La Jolla, CA, USA) was used to analyze the relationship between the substrate concentration and reactivity based on the Michaelis-Menten equation. SigmaPlot 12.0 software was used for the statistical analyses (Systat Software Inc., San Jose, CA). A paired t-test was used to analyse the samples of the two groups, and one-way ANOVA was used to test for differences among at least three groups. *P* < 0.05 was considered statistically significant.

## 3 Results

### 3.1 Optimization of peptide substrates

**Figure 2:**
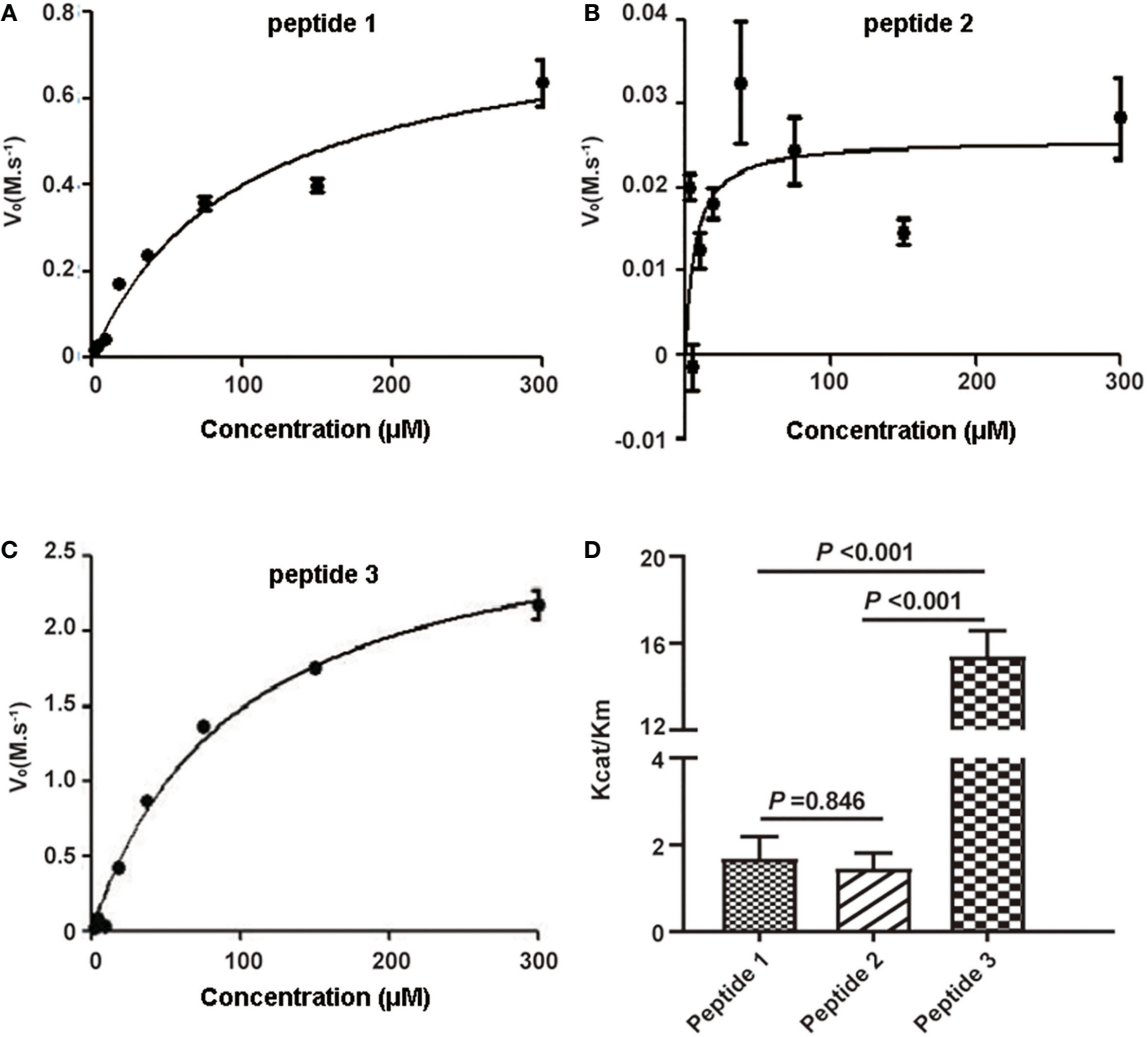
Kinetic analysis of human active C1s on cleavage of synthetic peptide substrates. Michaelis-Menten curves were generated where velocity (*v_o_*) was plotted against the concentrations of **(A)** peptide 1, **(B)** peptide 2, and **(C)** peptide 3; **(D)** K_cat_/K_m_ values of the proteolytic activity of C1s on three peptide substrates. Data are presented as the means ± standard deviation (n = 3). *P*-values were calculated by one-way ANOVA.

To select the optimal peptide substrate of active C1s for fluorescent quenched substrate assay, the kinetic parameters for C1s-mediated cleavage of the three candidate peptides were detected. Michaelis-Menten curves were generated, where initial velocity (*V_0_*) was plotted against substrate peptide 1 (**Figure 2A**), peptide 2 (**Figure 2B**), and peptide 3 (**Figure 2C**). The specificity for cleavage of substrate peptide 1, peptide 2, and peptide 3 by activated C1s were shown in **Figure 2D**. Kcat/Km value of peptide 3 was obviously higher than that of peptide 1 and peptide 2 (*P* < 0.001). However, Kcat/Km values were not significantly different between peptide 1 and peptide 2 (*P* = 0.846). Thus, peptide 3 was selected as the candidate substrate peptide for developing FRET-based fluorescent substrate peptide.

### 3.2 Determination of optimal reaction time and generation of standard curve

**Figure 3:**
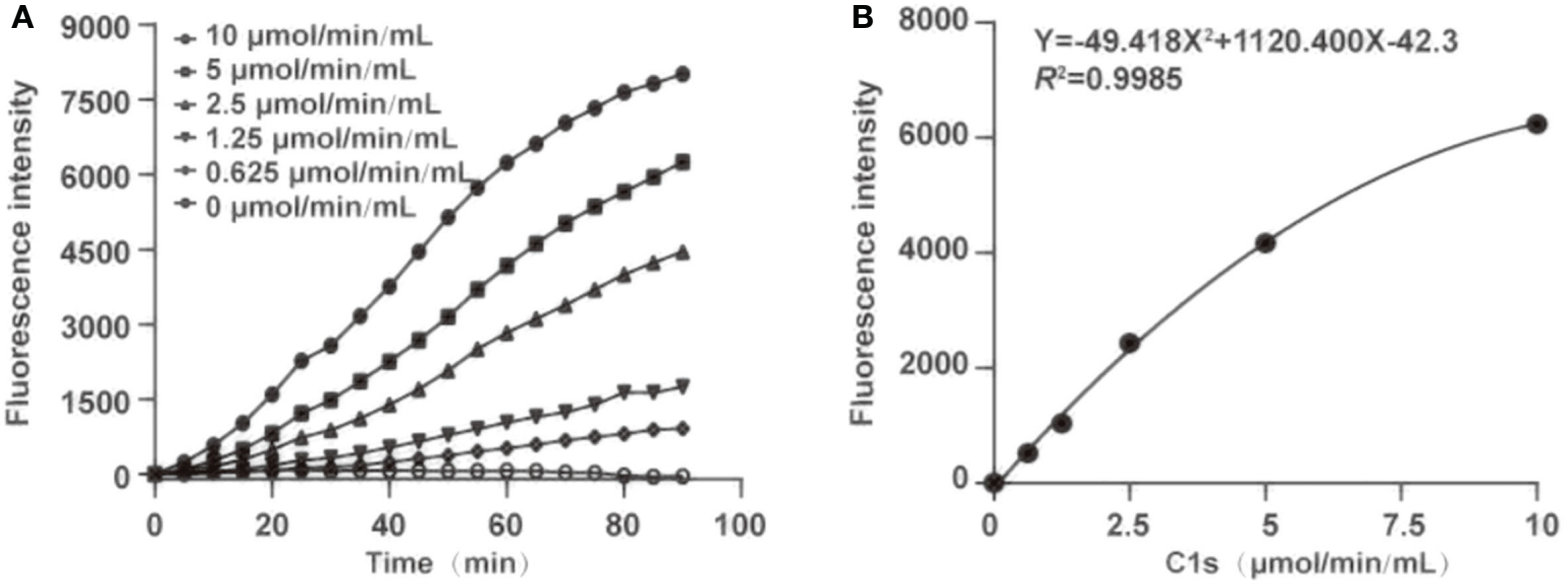
Optimization of the reaction time and generation of standard curve. **(A)** The optimum reaction time was ascertained in the presence of chromogenic peptide 3 (10 μL, 1 μg/mL). Fluorescence intensity was recorded at the indicated time; **(B)** Plot of standard curves were generated against activated C1s with different concentrations (0.625, 1.25, 2.5, 5, and 10 μmol·min^-1^·mL^-1^).

For determination of optimal reaction time, fluorescence intensity was recorded within 0-90 min (**Figure 3A**). Fluorescence intensity increased with the reaction time extended. Simultaneously, the fluorescence intensity was strongly increased by activated C1s with high enzyme activity. The correlation was preserved within 20-80 min by quadratic regression analysis. The R^2^ values of four reaction times were 0.984 ± 0.0114 (20 min), 0.993 ± 0.005 (40 min), 0.996 ± 0.003 (60 min), and 0.983 ± 0.005 (80 min), respectively (**Table 1**). As the R^2^ value at 60 minutes was closer to 1, this time was finally set as the optimal reaction endpoint. The standard curve of fluorescence and C1s activity was generated (**Figure 3B**). The following equation was obtained: Y = 49.418X^2^ + 1120.4X - 42.3 (R^2^ = 0.9985). **Figure 3B** showed that the fluorescence intensity increased with increasing C1s activity up to 10 μmol·min^-1^·mL^-1^.

**Table 1:** The *R* ^2^ value at various reaction times (*n* = 4).

Reaction time				
	20 min	40 min	60 min	80 min
*R* ^2^	0.984 ± 0.0114	0.993 ± 0.005	0.996 ± 0.003	0.983 ± 0.005

### 3.3 Determination of LOD

LOD was determined under optimal condition. The fluorescence intensity was repeatedly detected 20 mins for the reaction after the magnetic microbeads-labelled anti-C1s antibody was added with substrate peptide 3 in the absence of activated C1s. The LOD for activated C1s was 0.096 μmol·min^-1^·mL^-1^ in this immunoassay.

### 3.4 Accuracy and precision

Accuracy and precision of the method were then evaluated through recovery and repeatability. Prior to spiking, the activity of activated C1s in human serum samples was 0.246, 1.985, and 1.966 μmol·min^-1^·mL^-1^, respectively. **Table 2** showed that the recovery rates were 90.38%, 104.87%, and 90.34%, which was acceptable for the potential application of method in C1s detection in clinical samples. The intraday repeatability and interday reproducibility were examined. The results of intraday and interday assays were shown in **Table 3**. The CVs of repeatability and reproducibility were less than 10%, which clearly indicated an acceptable precision.

**Table 2:** Relative recovery of C1s obtained from human serum samples spiked with C1s of different activities.

Sample	C1s baseline (μmol min^-1^ mL^-1^)	C1s spiked (μmol min^-1^ mL^-1^)	C1s detected (μmol min^-1^ mL^-1^)	Recovery (%)
Sample 1	0.246	2.671	1.330	90.38
Sample 2	1.985	2.671	2.393	104.87
Sample 3	1.966	5.342	3.301	90.34

**Table 3:** Precision tests of C1s activity assay.

Samples	Intra-assay (*n* = 10)			Inter-assay (*n* = 10)		
	Means	SD	CV (%)	Means	SD	CV (%)
Low conc. (μmol·min^-1^·mL^-1^)	0.314	0.028	8.91	0.298	0.028	9.40
Med conc. (μmol·min^-1^·mL^-1^)	1.588	0.097	6.11	1.666	0.156	9.36
High conc.(μmol·min^-1^·mL^-1^)	4.567	0.305	6.68	4.412	0.374	8.48

### 3.5 Specificity

The cross-reaction rates of the C1r enzyme, MASP1 and MASP2 were all less than 0.5% with a rate of 0.15%, 0.04%, and 0.04%, respectively (**Table 4**). The cross-reaction with C1r enzyme, MASP1, and MASP2 was not significant, suggesting that the assay can specifically measure activated C1s in serum samples with high specificity. Cross-reactivity has been checked for C1r, MASP-1, MASP-2 (**Table 4**). The cross-reactivity measured for C1r seems quite high as compared to the others. This may be due to certain C1s contaminant in the C1r sample, which would contribute to the immunocapture and measured activity. Moreover, bilirubin, chyle, and haemoglobin in the clinical samples may potentially interfere with the assay for C1s activity. Therefore, the interference tests were further performed with activated C1s standard solutions at low, medium and high concentrations and interference substances including bilirubin (0, 0.06, 0.14, and 0.20 mg·mL^-1^), chyle (0, 600, 1400, and 2000 FTU), and haemoglobin (0, 1.5, 3.5, and 5 mg·mL^-1^). None of these interferents significantly affected the assay of C1s activity (**Table 5**).

**Table 4:** Cross-reactivity of C1s activity assay.

Interfering substances	C1s activity	Interference concentration	Cross-reactivity rate (%)
C1r enzyme	0.053 μmol·min^-1^·mL^-1^ (1 μg·mL^-1^)	660 μg·mL^-1^	0.15
MASP1	0.107 μmol·min^-1^·mL^-1^ (2 μg·mL^-1^)	5000 μg·mL^-1^	0.04
MASP2	0.096 μmol·min^-1^·mL^-1^ (1.8 μg·mL^-1^)	5000 μg·mL^-1^	0.04

**Table 5:** Interference of C1s activity assay by bilirubin, chyle and hemoglobin (*n* = 4).

	Low conc. (μmol·min^-1^·mL^-1^)	Med conc. (μmol·min^-1^·mL^-1^)	High conc. (μmol·min^-1^·mL^-1^)
Bilirubin conc. (mg·mL^-1^)			
0	0.323 ± 0.019	1.748 ± 0.166	3.322 ± 0.156
0.06	0.343 ± 0.019	1.822 ± 0.136	3.346 ± 0.269
0.14	0.335 ± 0.015	1.808 ± 0.077	3.258 ± 0.264
0.20	0.329 ± 0.025	1.581 ± 0.123	3.279 ± 0.185
Chyle conc. (FTU)			
0	0.339 ± 0.033	1.890 ± 0.130	3.321 ± 0.123
600	0.345 ± 0.018	1.942 ± 0.191	3.285 ± 0.135
1400	0.333 ± 0.040	1.739 ± 0.093	3.182 ± 0.317
2000	0.325 ± 0.056	1.992 ± 0.165	3.250 ± 0.323
Hb conc. (mg·mL^-1^)			
0	0.366 ± 0.025	1.800 ± 0.170	3.406 ± 0.241
1.5	0.352 ± 0.035	1.730 ± 0.068	3.555 ± 0.215
3.5	0.364 ± 0.033	1.653 ± 0.157	3.301 ± 0.269
5	0.374 ± 0.030	1.657 ± 0.085	3.493 ± 0.301

FTU, formazine turbidity unit; Hb, haemoglobin.

### 3.6 Effects of anticoagulants on activated C1s detection

**Figure 4:**
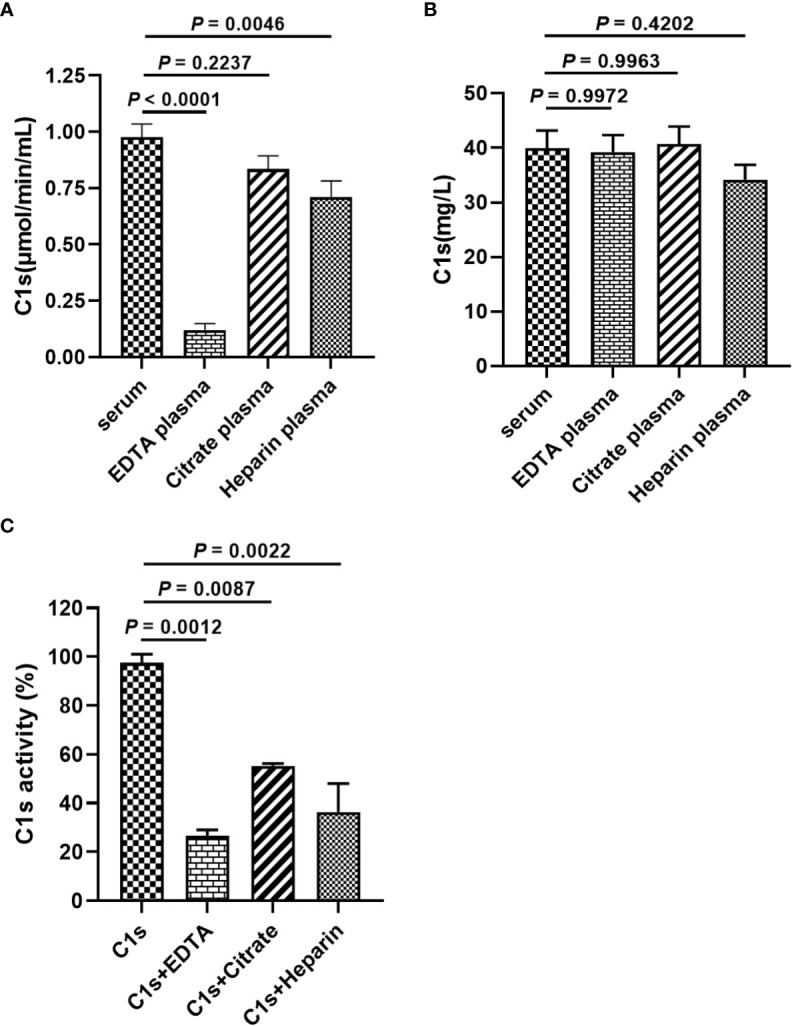
Effects of anticoagulants on FRET-based immunoassay for activated complement C1s detection. **(A)** Enzyme activity of complement C1s in serum and plasma samples by FRET-based immunoassay; **(B)** Protein contents of C1s in serum and plasma samples by ELISA; Human blood samples (n = 18) were collected with EDTA, citrate and heparin as anti-agglutinins. **(C)** Activated C1s standard solutions assayed by FRET-based immunoassay after treatment with EDTA, citrate and heparin; Data are presented as the means ± standard deviation (n = 3). ns, P>0.05; **P<0.01; ***P<0.001; P<0.0001. *P*-values were calculated by one-way ANOVA.

Anticoagulants are required for blood collection and isolation to inhibit blood clotting. It is unclear whether anticoagulants used during blood collection interfere with the cleavage of substrate peptide by activated C1s. **Figure 4A** showed that the enzymatic activities of complement C1s were significantly decreased after EDTA (*P* < 0.0001) or heparin (*P* < 0.01) treatment. Although the *p* values between citrate and serum was less significant (*P* > 0.05), citrate treatment also showed some degree suppressive effects on the cleavage of C1s in plasma samples. The total protein levels of C1s were also detected by ELISA, and the data in **Figure 4B** showed that no significant differences in C1s contents between serum and three anticoagulants-treated groups (*P* > 0.05). Next, to confirm the effects of anticoagulants on the enzymatic activities of activated C1s, the activated C1s standard solutions (25 pmol·min^-1^·μg^-1^) were treated with EDTA (1.5 mg·mL^-1^), citrate (109 mmol·L^-1^), and heparin (15 IU·mL^-1^). The data in **Figure 4C** showed that the enzymatic abilities of C1s were suppressed by EDTA (26.54%) (*P* < 0.001), citrate (55.27%) (*P* < 0.01), and heparin (36.4%) (*P* < 0.01). Thus, these data demonstrated that the assayed anticoagulants, especially EDTA, significantly suppressed the enzymatic activity of activated C1s in the clinical samples.

### 3.7 Determination of activated C1s in clinical serum samples

**Figure 5:**
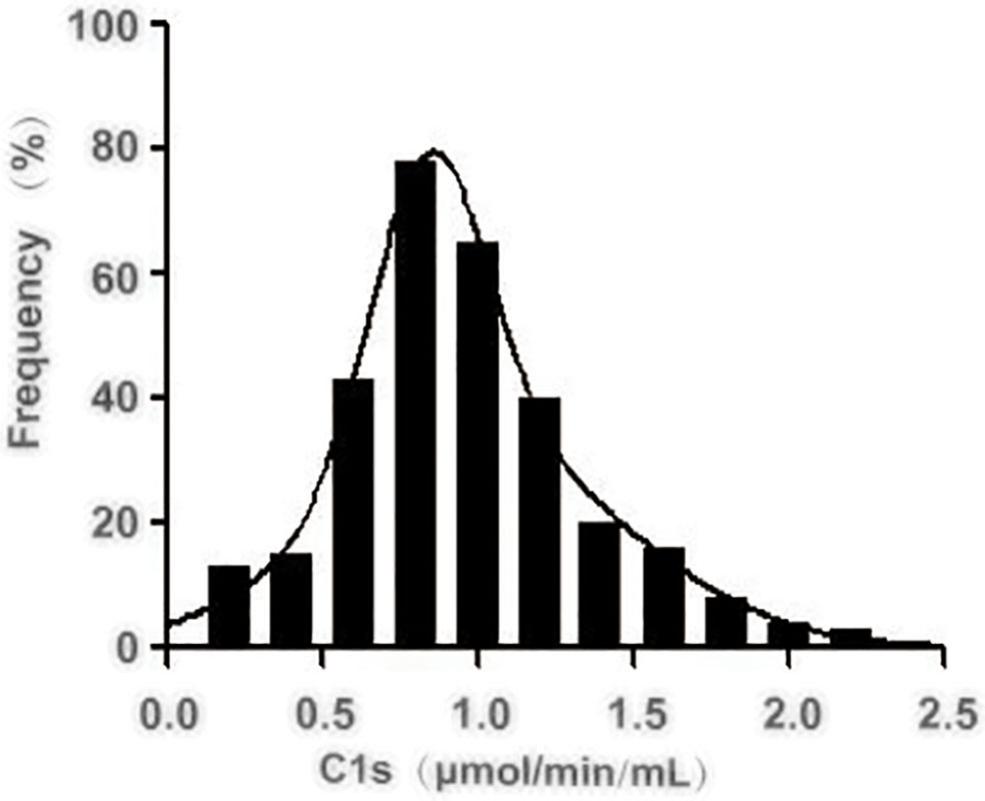
Distribution of the activated complement C1s activity in serum from healthy individuals. The activity of C1s was analysed by FRET-based immunoassay. n = 306.

Next, we investigated the reference activity range of activated C1s in the healthy individuals by the established FRET-based immunoassay. A total of 306 healthy individuals were included in the analysis, and the corresponding serum samples were collected for the quantitative analysis of C1s activity. The obtained results were shown in **Figure 5** and **Table 6**. The minimum and maximum values of the C1s activity in the detected samples were 0.165 and 2.547 μmol·min^-1^·mL^-1^. The mean value and standard deviation were 1.023 μmol·min^-1^·mL^-1^ and 0.422 μmol·min^-1^·mL^-1^. The central 94.77% healthy individuals (290/306) were detected with activated C1s in the range of 0.26 and 1.90 μmol·min^-1^·mL^-1^, indicating that the reference range of activated C1s assayed by FRET-based immunoassay was 0.26-1.90 μmol·min^-1^·mL^-1^. However, this reference range was obtained based on a small sample cohort. In future studies, the sample size will be expanded and the significance of the established FRET-based immunoassay will be explored in a variety of diseases.

**Table 6:** Reference interval of activated C1s in healthy individuals (n=306).

	Activated C1s (μmol·min^-1^·mL^-1^)					
	< 0.260	0.261-0.670	0.671-1.080	1.081-1.490	1.491-1.900	> 1.900
Samples	8	55	144	65	26	8
Percentage (%)	2.61	17.97	47.06	21.24	8.50	2.61
Total (%)	2.61	20.59	67.65	88.89	97.39	100

### 3.8 Determination of active C1s in serum samples from patients with RA

**Figure 6:**
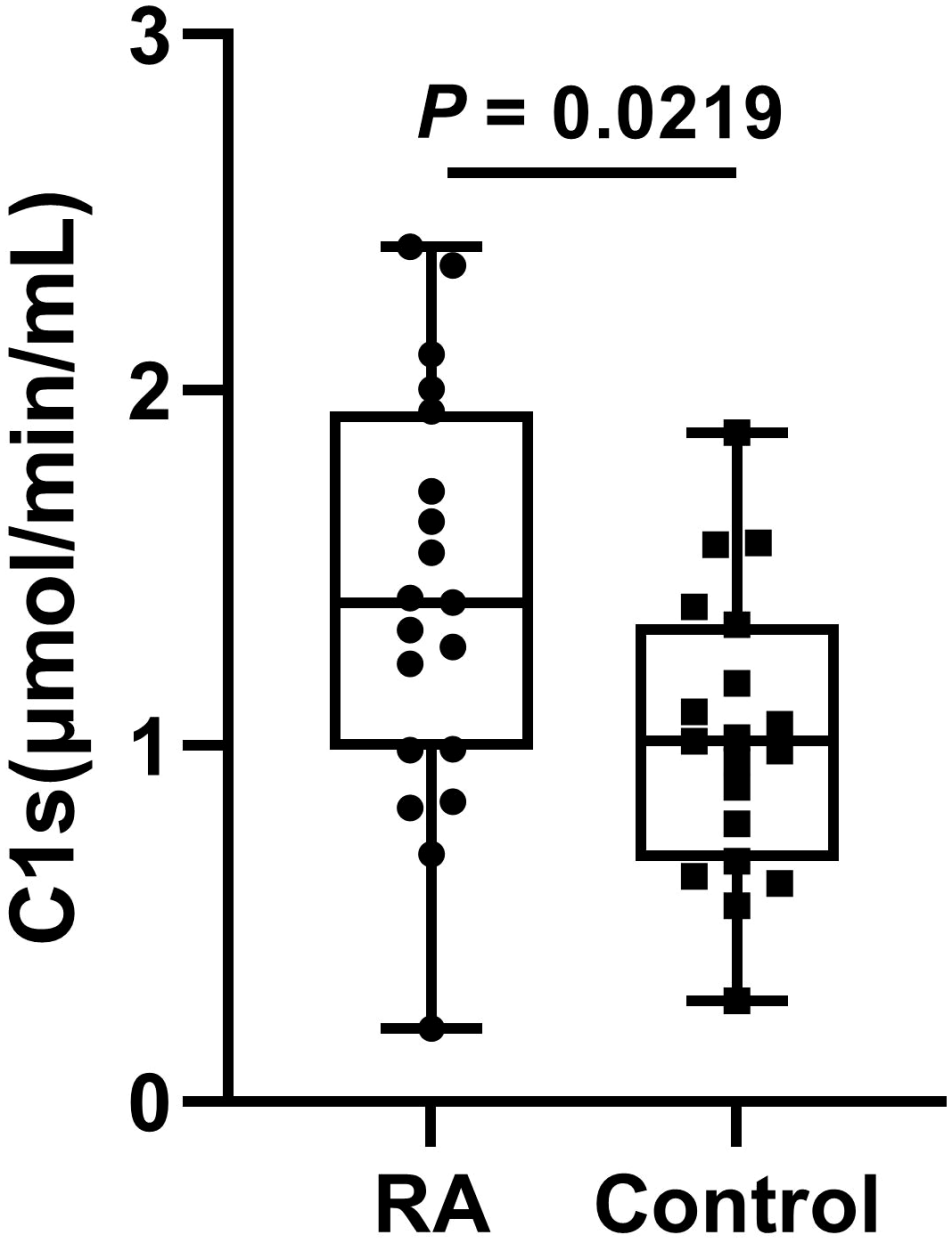
The levels of active C1s in serum of patients with RA. Data are presented as the means ± standard deviation (each group: n = 20). *P* = 0.0219 by *t* test.

The serum samples of 20 patients with RA and 20 healthy participants were measured. The results showed that the active C1s in the serum samples from patients with RA was significantly increased compared to that of the control group (*P* < 0.05) (**Figure 6**).

## 4 Discussion

The detection methods for C1s reported include bilateral diffusion (14), ELISA, gelatin zymography (15) and LC-MS/MS (16). However, bilateral diffusion, gelatin zymography, and LC-MS/MS techniques only enable qualitative or semi-quantitative analysis. The accuracy of the results is poor and the CV values are highly variable as the ELISA plate need to be repeatedly cleaned during the assay. More notably, these methods only measure the level of C1s protein and are unable to distinguish between C1s being in the active or inactive state. C1s are normally presented *in vivo* as zymogens and exert enzymatic activity when activated by C1r. Therefore, accurate determination of activated C1 activity is particularly important for understanding the exact role of C1 in physiological and pathological conditions in humans and mammals.

A previous study had determined the active status of C1s exploiting the characteristic of C1s cleaved into C2 and C4 (23). Unfortunately, the procedure of this method is cumbersome to perform. Additionally, this method shows limited specificity as the targets C4 and C2 molecules can also be cleaved by MASP2. In certain cases, DTNB was employed to measure the activity of recombinant C1s (27). Unfortunately, DTNB also serves as a substrate for granzyme H (26). Thus, DTNB are not suitable for measuring C1s activity in a specific manner. The assessment of complement function has been designed based on the regulatory effects of C1 inhibitor on the proteases of the C1 complex (C1r and C1s) (28). However, this method is not sufficient because it not only relies on the convenient spectrophotometric measurement of the active C1s, but also the activated C1r. Thus, it could be used for C1INH detection, but it is not available for active C1s assay due to lack of specificity.

The subcomponent C1s in the serum acts as the pro-enzymic form and is responsible for the activation of components C2 and C4 (29–31). Based on this functional mechanism, the unique serine protease activity of activated C1s, which can specifically cleave the target proteins C2 and C4, was combined with FRET technology to detect C1s activity. Here, specific peptides were designed considering that active C1s catalyzes the cleavage of C2 between R243 and K244 (peptide 1), and C4 between R756 and A757 (peptide 2) (24, 25). Next, FRET-based fluorogenic peptides 3 were synthesized, and the activity of active C1s was indicated by calculating the Kcat/Km value. The results showed that the Kcat/Km values of peptide 3 were significantly higher than the values of peptide 1 and peptide 2. FRET-based peptide 3 was selected as a candidate substrate for the detection of active C1s in serum samples. The active C1s in the system cleave the substrate peptides, and then Abz would no longer be quenched by Dnp. Consequently, chromogenic peptide could emit detectable fluorescence signals. However, if active C1s are absent in the system, the substrate peptides remain intact and the distance between Abz and Dnp is 10-100 angstroms (Å). Therefore, Dnp quenches the fluorescence emitted by Abz, and the fluorescence could not be detected. The fluorescence intensity based on FRET is strongly related to the activity of active C1s (**Figure 1**).

It is noteworthy that MASP-2 is another key enzyme that can cleave C4 and C2 to assemble C3 convertase (C4b2a), which may interrupt the specificity in clinical samples (32). Also within the realm of possibility is the fact that the selected substrate peptide 3 can be cleaved by other protease. Hence, in this study, magnetic microbeads were conjugated with recombinant C1s-specific antibody that could specifically capture active C1s, and this complex can be magnetically separated from the interfering mediums. Subsequently, we established a FRET-based immunoassay to detect active C1s, which showed low detection limits, good correlation, wide linearity range and acceptable recoveries (**Figures 1**, **2**). In particular, this assay method we developed is effective in avoiding interference from bilirubin, chyle and haemoglobin. As for the intra-assay CV, it has been indicated that the intra-assay CV for assaying the activity of the classical pathway, alternative pathway, and mannose-binding lectin ranged between 35% and 99%, while the CVs of the two controls for C1INH activity were 43% and 28%, respectively (33). These results indicate that there are uncertain influences in the current assays for complement activity and single complement component. In the present study, we controlled the CVs of repeatability and reproducibility of active C1s within 10%.

The C1 complex is a calcium-dependent complex composed of three distinct subcomponents (34). Both EDTA and citrate can bind calcium ions, and little is known about the effects of these anticoagulants on the activity of classic complement pathway initiated by the C1 complex. In our study, we observed that heparin, EDTA, and citrate significantly reduce the activity of classical, alternative and mannose-binding lectin pathways, with only about 50% of classic complement pathway activity in plasma treated with anticoagulants (heparin, EDTA, and citrate) compared to complement activity in serum (**Figure 4**). Another study showed that C1 hemolytic activity was significantly reduced in a dose-dependent manner in both EDTA- and citrate-treated samples. Subsequent hemolytic activity of C1 could not be restored even after recalcification (35). Ca^2+^ binding is the premise of C1s activation, which indicates that Ca^2+^ also plays an important role in regulating C1s function. Actually, C1s zymogen and active C1s with catalytic ability have comparable binding valence to Ca^2+^ and similar affinity. This binding causes the generation of a dimeric molecule of 6.0s from a 4.5s monomer, which is a dimerization reaction that appears to primarily involve the chain of each monomer, presumably with these two Ca^2+^ binding sites located on the A chain of the C1s dimer (34). Heparin is another anticoagulant frequently available in clinical practice, which has been proved to affect C1s activity by binding to the SP domain of C1s and facilitating C1s biding to C1IHN (36, 37). Recently, our preliminary data showed that heparin or EDTA can significantly inhibit C1s activity in plasma compared to serum. Furthermore, no differences in C1 protein levels were observed in samples treated with heparin, EDTA or citrate, respectively.

RA is an autoimmune disease. It has been shown that the activation of the classical pathway is present in the serum of patients with RA (38). Moreover, serum samples from RA showed significantly elevated levels of C5b6789 (39). Assayed with our established method, active C1s in the serum samples is significantly elevated, suggesting activation of the classical pathway in patients with RA (**Figure 6**).

In summary, we established a FRET-based immunoassay to detect active C1s. This method is suitable for the detection of active C1s in serum samples within the reference range. In complement-related diseases, the levels of active C1s in serum are fundamental information for the diagnosis and treatment of diseases as well as for the study of its pathogenesis.

## Data availability statement

The original contributions presented in the study are included in the article/**Supplementary Material**. Further inquiries can be directed to the corresponding authors.

## Ethics statement

The studies involving human participants were reviewed and approved by the Ethics Committee of Taizhou People’s Hospital (KY2020-184-01). The patients/participants provided their written informed consent to participate in this study.

## Author contributions

SX and LZ contributed to conception and design of the study. JY and JX organized the database. JY and CZ performed the statistical analysis. JY wrote the first draft of the manuscript. JX, CZ, LZ and SX wrote and revised the manuscript. All authors contributed to the article and approved the submitted version. 
