# Supplementary material for: Quantitative fluorescence resonance energy transfer-based immunoassay for activated complement C1s
Source: Front Immunol. 2023 Jan 24;14:1081793. doi: 10.3389/fimmu.2023.1081793 (PMC9904206; doi:10.3389/fimmu.2023.1081793)
Supplement: Supplementary file 1 [file DataSheet_1.pdf]

Supplementary Figure 1. Recombinant C1s antibody was identified.

Recombinant anti-C1s was characterized by polyacrylamide gel electrophoresis and size exclusion chromatography-high performance liquid chromatography (SEC-HPLC). The data in Figure 1A indicated there were two protein bands in the reductive gel, and only one protein band in the non-reductive gel. The results from SEC (Figure 1B) showed the purity of anti-C1s is 95.5%.

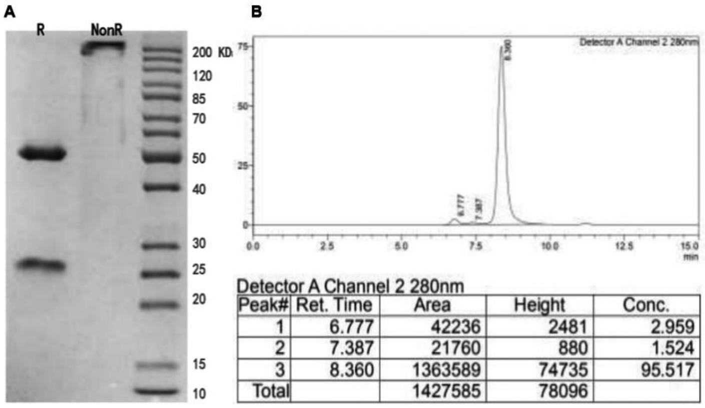

Detector A Channel 2 280nm

Peak#	Ret. Time	Area	Height	Conc.
1	6.777	42236	2481	2.959
2	7.387	21760	880	1.524
3	8.360	1363589	74735	95.517
Total		1427585	78096	

A: Recombinant anti-C1s was detected by using polyacrylamide gel electrophoresis; B: Recombinant anti-C1s was analyzed by using molecular exclusion chromatography-high performance liquid chromatography (SEC-HPLC).

R: reductive polyacrylamide gel electrophoresis; NonR: non-reductive polyacrylamide gel electrophoresis)
