# Supplementary material for: Quantitative fluorescence resonance energy transfer-based immunoassay for activated complement C1s
Source: Front Immunol. 2023 Jan 24;14:1081793. doi: 10.3389/fimmu.2023.1081793 (PMC9904206; doi:10.3389/fimmu.2023.1081793)
Supplement: Supplementary file 2 [file DataSheet_2.pdf]

Supplementary Figure 2. The binding ability of recombinant anti-C1s with active C1s (free), C1 and C1s zymogen.

The antibody captures abilities for C1s zymogen, activated C1s (free) and C1 complex (C1q2C1r2C1s) were analyzed by ELISA, which was desecribed in detail as below. The results showed that the C1s antibody had stronger activity to bind to active C1s (free) than to bind to C1 or C1s zymogen. However, there were no binding between C1s antibody and C1r.

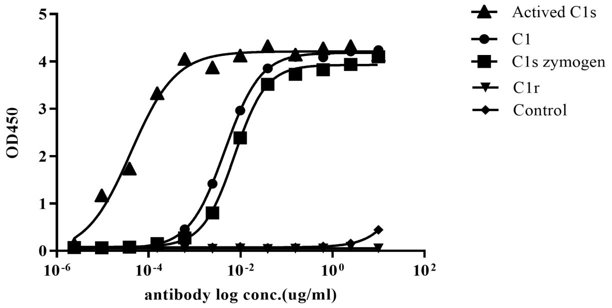

ELISA plates were coated with 2 μg/mL of active C1s, C1 complex (C1), C1s zymogen, respectively. Then, recombinant C1s monoclonal antibody and HRP-labeled secondary antibody were sequentially added into wells. Finally, chromogenic substrate were added and the absorbance values at OD450 were measured.
