# Supplementary material for: Quantitative fluorescence resonance energy transfer-based immunoassay for activated complement C1s
Source: Front Immunol. 2023 Jan 24;14:1081793. doi: 10.3389/fimmu.2023.1081793 (PMC9904206; doi:10.3389/fimmu.2023.1081793)
Supplement: Supplementary file 3 [file DataSheet_3.pdf]

Supplementary Figure 3. Effects of anti-C1s on the enzymatic ability of active C1s to cleave substrate peptide

To investigate the effect of different concentrations of C1s antibody on C1s enzyme activity to cleave substrate peptide 3, C1s antibodies with different concentrations (0, 5, 10, 20, 40, 80 and 160 μg/mL) were used to incubate with active C1s, followed by the addition of substrate peptide. The results showed that the addiation of C1s antibody had no obvious suppressive effects on C1s enzyme activity to cleave substrate peptide 3.

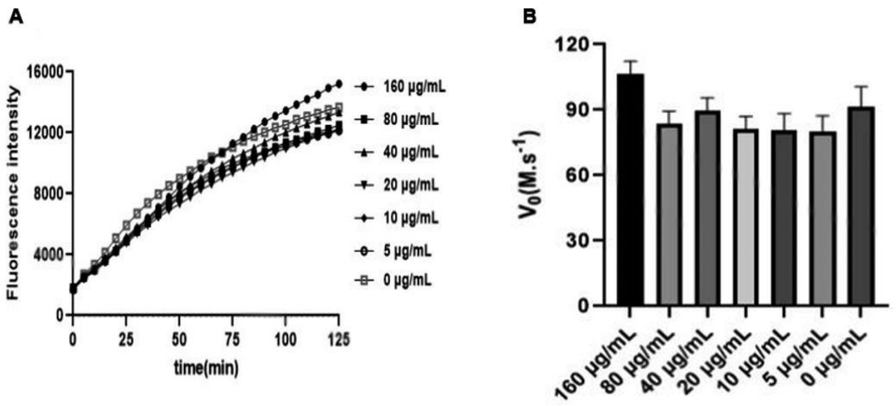

A: Effects of antibodies with different concentrations on the fluorescence intensity from substrate peptide cleaved by C1s;

B: Effect of antibodies with different concentrations on the kinetics of cleavage of the peptide substrate by C1s.
